# Elucidation of the Activation Pathways of ScyA1/ScyR1, an Aco/ArpA-Like System That Regulates the Expression of Nemadectin and Other Secondary Metabolic Biosynthetic Genes

**DOI:** 10.3389/fbioe.2020.589730

**Published:** 2020-11-03

**Authors:** Hui Liu, Yanyan Zhang, Shanshan Li, Jiabin Wang, Xiangjing Wang, Wensheng Xiang

**Affiliations:** ^1^School of Life Sciences, Northeast Agricultural University, Harbin, China; ^2^State Key Laboratory for Biology of Plant Diseases and Insect Pests, Institute of Plant Protection, Chinese Academy of Agricultural Sciences, Beijing, China

**Keywords:** nemadectin, activation pathway, ScyA1/ScyR1, *Streptomyces cyaneogriseus* ssp. *noncyanogenus*, autoregulator synthase, quorum-sensing system

## Abstract

The quorum-sensing system, consisting of an autoregulator synthase (AfsA or Aco homolog) and an autoregulator receptor (ArpA homolog), has been reported to be universally involved in regulating secondary metabolism in streptomycetes. Although the autoregulator synthase is thought to activate antibiotic production, the activation pathway remains poorly understood. *Streptomyces cyaneogriseus* ssp. *noncyanogenus* NMWT1 produces nemadectin, which is widely used as a biopesticide and veterinary drug due to its potent nematocidal activity. Here, we identified the Aco/ArpA-like system ScyA1/ScyR1, the ArpA homolog ScyR2 and the AfsA/ArpA-like system ScyA3/ScyR3 as important regulators of nemadectin production in NMWT1. Genetic experiments revealed that these five genes positively regulate nemadectin production, with *scyA1* and *scyR1* having the most potent effects. Importantly, ScyA1 is an upstream regulator of *scyR1* and promotes nemadectin production and sporulation by activating *scyR1* transcription. Intriguingly, *scyR1* silencing in NMWT1 up-regulated 12 of the 17 secondary metabolite biosynthetic core genes present in the NMWT1 genome, suggesting that ScyR1 mainly to be a repressor of secondary metabolism. In conclusion, our findings unveiled the regulatory pathways adopted by the quorum-sensing system, and provided the basis for a method to enhance antibiotic production and to activate the expression of cryptic biosynthetic gene clusters.

## Introduction

*Streptomyces* species produce various bioactive compounds with diverse effects, including immunosuppressive, antibacterial, antiviral, antitumor, insecticidal, and antiparasitic effects. Secondary metabolites from *Streptomyces* are widely used in veterinary, agricultural and medical areas (Liu et al., 2013; Guan et al., 2019). Notably, approximately 70% of the commercially available antibiotics are derived from *Streptomyces* (Kitani et al., 2011). The genome of *Streptomyces* also contains numerous secondary metabolite gene clusters that are cryptic or expressed at low levels (Liu et al., 2013). Despite their low expression, these silent gene clusters are critical for natural product discovery. The expression of secondary metabolite gene clusters is stringently controlled by complex cellular regulatory network, in which regulatory proteins of different families coordinate with environmental and physiological cues (Zhou et al., 2020). The quorum-sensing (QS) system components, including autoregulator synthase (AfsA or Aco homolog) and autoregulator receptor (ArpA homolog), have been identified as important regulators of secondary metabolism and development in most streptomycetes (Kitani et al., 2011; Niu et al., 2016). Thus far, 36 autoregulators have been identified, which based on their structure can be classified into five groups: γ-butyrolactones (GBLs), butenolides, furans, PI factors, and *N*-methylphenylalanyl-dehydrobutyrine diketopiperazine (Niu et al., 2016; Wang et al., 2018). However, only two types of autoregulator synthases have been identified, the AfsA-like proteins that produce GBLs, furans, or butenolides, as well as Aco-like synthases that produce butenolides (Kitani et al., 2011; Wang et al., 2018). The majority of autoregulator receptors are ArpA-like proteins of TetR family. Therefore, based on the synthase type, QS systems can be classified into AfsA/ArpA-type and Aco/ArpA-type.

In most cases, the autoregulator synthase is essential to initiate antibiotic production (Zou et al., 2014). Thus, deciphering the pathway it activates secondary metabolism, particularly the mechanisms through which it cooperates with the autoregulator receptor to control production of secondary metabolites has become one of the main parts in the research of natural products biosynthesis. To date, the reported well-known activation way is that, with the accumulation of autoregulators produced by the synthase, autoregulators of sufficient concentration bind to the cognate receptor ArpA, release the repression of ArpA to its targets, and activate the biosynthesis of secondary metabolites through a regulatory cascade (Niu et al., 2016). This regulatory means has been perfectly interpreted in *Streptomyces griseus*, *Streptomyces ansochromogenes*, and *Streptomyces avermitilis*. In *S. griseus*, A-factor is essential for removing the inhibition of ArpA on the transcription of *adpA*, whose protein product then activates *strR* transcription, initiating streptomycin biosynthesis (Horinouchi, 2007). Similarly, in *S. ansochromogenes*, The SAB signals, produced by the autoregulator synthase SabA, dissociate SabR1 from *cprC* promoter, activating the CprC/AdpA/SanG pathway and nikkomycin production (Wang et al., 2018). In *Streptomyces avermitilis*, the avenolide-like autoregulator synthase Aco is required for avermectin production; both AvaR1 and AvaR2 are the receptors of avenolide and the direct repressors of avermectin production; the avenolides could relieve the direct repression of AvaR1 and AvaR2 in control of avermectin production (Kitani et al., 2011; Zhu et al., 2016, 2017). However, this activation mechanism might only be one of the diverse strategies by which autoregulator synthase triggers antibiotic production. A report by Zou and coworkers showed that the autoregulator synthases (*jadW1*/*W2*/*W3*) are essential for jadomycin production, the autoregulator receptor JadR3 has also been shown to be an activator of jadomycin biosynthesis; thus, the mechanisms by which JadW1/W2/W3 promotes jadomycin production remain unknown (Zou et al., 2014).

Nemadectin, a 16-membered macrolide antibiotic produced by *Streptomyces cyaneogriseus* ssp. *noncyanogenus*, is mainly used for the semi-synthesis of moxidectin (Li C. et al., 2019), a methoxime derivative of nemadectin. Moxidectin is often used as a veterinary drug to eliminate nematodes and external parasites and has been proposed as a treatment for human scabies (Mounsey et al., 2017). Since 2018, moxidectin is also used to treat river blindness caused by *Onchocerca volvulus* in patients ≥12 years old. Given the great commercial value of nemadectin and moxidectin, the development of methods to increase production yield and reduce production cost is of high importance. Additionally, elucidating the regulatory networks underlying antibiotic biosynthesis will facilitate the genetic engineering of *Streptomyces* to generate novel high-producing strains (Li D. et al., 2019; Li et al., 2020). However, the genetic regulators controlling nemadectin biosynthesis remain largely unknown except for one confirmed cluster-situated activator NemR (Li C. et al., 2019), hindering the rational design of nemadectin hyper-producer strains using regulator-based strategies.

In this study, we investigated the regulatory role of QS systems in nemadectin biosynthesis in *S. cyaneogriseus*. We found that the *aco/arpA*-like system (*TU94_985/TU94_975*, hereafter referred to as *scyA1*/*scyR1*), the putative *arpA* homolog (*TU94_3165*, *scyR2*), and the *afsA*/*arpA*-like system (*TU94_11455/TU94_11460*, *scyA3*/*scyR3*) promoted nemadectin production. In particular, *scyA1* and *scyR1* strongly affected morphological differentiation and nemadectin production in *S. cyaneogriseus.* We also found that ScyA1 was essential for *scyR1* transcription, thereby promoting sporulation and nemadectin production. Additionally, ScyA1 activated the expression of many other secondary metabolite biosynthetic core genes, whereas ScyR1 had the opposite effect. Importantly, *scyR1* silencing enhanced the expression of numerous biosynthetic core genes. These data provide further insight into the role of the QS system in secondary metabolism in *S. cyaneogriseus* and provide new ways to increase antibiotic production and to activate the silent biosynthetic gene clusters.

## Materials and Methods

### Strains, Plasmids and Growth Conditions

All strains and plasmids used in this study are summarized in Supplementary Table 1. The nemadectin producer *Streptomyces cyaneogriseus* ssp. *noncyanogenus* NMWT1 has been deposited at Agricultural Research Service Culture Collection (accession No. NRRL 15773). Escherichia coli was cultured in Luria Bertani (LB) medium supplemented with antibiotics as required at 37°C. *S. cyaneogriseus* ssp. *noncyanogenus* NMWT1 and its derivatives were grown on ISP3 agar medium with/without apramycin at 37°C for sporulation. Flask fermentation of *S. cyaneogriseus* ssp. *noncyanogenus* strains was performed as described previously (Li C. et al., 2019).

### Primers

All primers used in this study are summarized in Supplementary Table 2.

### Gene Deletion, Complementation and Overexpression

Deletion experiments of *scyA1*, *scyR1*, *scyR2*, *scyA3*, and *scyR3* were performed individually in *S. cyaneogriseus* ssp. *noncyanogenus* NMWT1 by the CRISPR/Cas9-mediated genome-editing system (Huang et al., 2015). Briefly, the five target-specific guide RNAs (*scyA1*-sgRNA, *scyR1*-sgRNA, *scyR2*-sgRNA, *scyA3*-sgRNA, and *scyR3*-sgRNA) designed for construction of the disruption mutants were amplified from pKCcas9dO (Addgene No. 62552) (Supplementary Table 1); left and right fragments flanking each gene were amplified from *S. cyaneogriseus* ssp. *noncyanogenus* NMWT1 genomic DNA. All primers were listed in Supplementary Table 2. Then the PCR products were ligated into the corresponding sites of pKCcas9dO digested by *Spe*I/*Hin*dIII, generating pKCcas9dscyA1, pKCcas9dscyR1, pKCcas9dscyR2, pKCcas9dscyA3, and pKCcas9dscyR3 (Supplementary Table 1). All the deletion plasmids were transferred separately into *E. coli* ET12567/pUZ8002 and then introduced into *S. cyaneogriseus* ssp. *noncyanogenus* NMWT1 via intergenic conjugation (Kieser et al., 2000). Guided by sgRNAs, the Cas9 cut the chromosomal sites. By homologs chromosomic recombination with linearized fragments, the targeted genes were disrupted. Finally, using this method, we obtained four genes’ mutant strains, which were further confirmed as deletion mutants (ΔscyA1, ΔscyR2, ΔscyA3, and ΔscyR3) by PCR and DNA sequencing.

*scyR1* repression strain was constructed in *S. cyaneogriseus* ssp. *noncyanogenus* NMWT1 using CRISPR/dCas9-based interference system (CRISPRi) (Zhao et al., 2018). In brief, the *scyR1* specific single guide RNA (d*scyR1*-sgRNA) designed for *scyR1* repression was amplified from pSET-dCas9 using primer pairs CRISPRi-scyR1sgRNAF/R (Supplementary Table 2). The amplified product of sgRNA was inserted into the plasmid pSET-dCas9 carrying the dCas9 expression cassette. The resulting plasmid (pSET-dCas9-*scyR1*) was introduced into *S. cyaneogriseus* ssp. *noncyanogenus* NMWT1 by conjugation. The desired mutant (RscyR1) was further confirmed by quantitative real-time RT-PCR (qRT-PCR) analysis.

For overexpression of the five genes (*scyA1*, *scyR1*, *scyR2*, *scyA3*, and *scyR3*) in *S. cyaneogriseus* ssp. *noncyanogenus* NMWT1, the fragments containing promoterless genes were amplified from *S. cyaneogriseus* ssp. *noncyanogenus* NMWT1 genomic DNA; the *hrdB* promoter (P_hrdB_) was amplified with primer pairs hrdB-pF/R. Then these target gene open reading frames (ORFs) controlled by P_hrdB_ were cloned into pSET152 using the ClonExpress^TM^ MultiS One Step Cloning Kit (Vazyme) to obtain the overexpression plasmids (pSET152-P_hrdB_scyA1, pSET152-P_hrdB_scyR1, pSET152-P_hrdB_scyR2, pSET152-P_hrdB_scyA3, and pSET152-P_hrdB_scyR3) (Supplementary Table 1). The resulting plasmids were introduced into *S. cyaneogriseus* ssp. *noncyanogenus* NMWT1 via *E. coli*–*Streptomyces* conjugation (Kieser et al., 2000), respectively, generating OscyA1, OscyR1, OscyR2, OscyA3, and OscyR3, respectively (Supplementary Table 1). pSET152-P_hrdB_scyA1 and pSET152-P_hrdB_scyR1 were also introduced into ΔscyA1, generating ΔscyA1/P_hrdB_scyA1 and ΔscyA1/P_hrdB_scyR1, respectively (Supplementary Table 1).

### Fermentation and HPLC Analysis of Nemadectin

Nemadectin fermentation and detection conditions were the same as previous report (Li C. et al., 2019).

### Protein Expression and Purification

The proteins of ScyR1 and ScyR3 were expressed with His_6_ labels and ScyR2 was expressed with a label of GST. The coding regions were amplified by PCR using respective primer pairs listed in Supplementary Table 2. PCR products of *scyR1* and s*cyR3* were separately digested with *Nde*I and *Xho*I and then inserted into the corresponding site of pET-23b (+), generating pET-23b:scyR1 and pET-23b:scyR3 (Supplementary Table 1), respectively; PCR products of *scyR2* were digested with *Eco*RI and *Xho*I, and then cloned into pGEX-4T-1, generating pGEX-4T-1:scyR2 (Supplementary Table 1). All the expression plasmids were verified by nucleotide sequencing and then introduced into *E. coli* BL21 (DE3), respectively. Purification and protein concentrations were performed as described previously (He et al., 2018; Li C. et al., 2019).

### Electrophoretic Mobility Shift Assays (EMSAs)

Electrophoretic mobility shift assays (EMSAs) were performed as reported previously (Li C. et al., 2019). The promoter probes were obtained by PCR from the genomic DNA of *S. cyaneogriseus* ssp. *noncyanogenus* NMWT1 with primer pairs listed in Supplementary Table 2.

### RNA Extraction and Quantitative Real-Time RT-PCR Analysis

The fermentation cultures of *S. cyaneogriseus* ssp. *noncyanogenus* NMWT1 and its derivatives were collected at various time points (0.75, 2, 3, and 6 days), respectively. RNA extraction, removal of genomic DNA, examination of RNA quality and concentration, synthesis of cDNA and qRT-PCR were performed as described previously (Zhang et al., 2016). Primers used for qRT-PCR were listed in Supplementary Table 2.

### GFP Reporter Assay in *E. coli*

To construct the green fluorescence gene (*gfp*) reporter plasmids, the original plasmid pSET152 was cut with *Bam*HI and *Xba*I. The *scyA1* promoter (P_A1_) and the coding region of *scyR1* were amplified from *S. cyaneogriseus* ssp. *noncyanogenus* NMWT1 genomic DNA with primer pairs pscyA1GFPF/R and ScyR1GFPF/R, respectively. The *gfp* and the strong constitutive promoter SF14 were amplified from pSET152:P*_sbbA_gfp*:SF14*sbbR* with primer pairs GFPF/R and pSF14F/R, respectively (He et al., 2018). First, P_A1_ and *gfp* coding region were ligated into pSET152 using the ClonExpress^TM^ MultiS One Step Cloning Kit (Vazyme) to obtain pSET152:P_A1_*gfp* (Supplementary Table 1). Secondly, the plasmid pSET152:P_A1_*gfp* was digested with *Nhe*I and then assembled with SF14 promoter and the *scyR1* coding region to generate the corresponding reporter plasmid pSET152:P_A1_*gfp*:SF14*scyR1* (Supplementary Table 1), in which *scyR1* was controlled by SF14 and the *gfp* gene was controlled by P_A1_. These two plasmids together with the control vector pSET152 were introduced into DH5α, respectively, to detect the intensity of green fluorescence (excitation at 485 nm; emission at 535 nm, Synergy H4 Multi-Mode Reader). All fluorescence values were normalized to growth rates (OD_600_).

### Preparation of Autoregulator Culture Extracts

The procedure for preparation of autoregulator culture extracts in this study was as described previously with minor modification (Kitani et al., 2011; Zou et al., 2014). A total of 300 mL seed culture broth of *S. cyaneogriseus* ssp. *noncyanogenus* NMWT1 or ΔscyA1 strain cultured in seed medium for 2 days was extracted with equal volumes of ethyl acetate. The organic phase was dried in a vacuum rotary evaporator and re-dissolved in 1 mL DMSO.

### Nucleotide Sequence Accession Number

The ∼8.5 kb DNA fragment containing *TU94_00970*, *TU94_00975*, and *TU94_00985* was re-sequenced and deposited under the GenBank accession No. MT563325.

### Statistical Analysis

All experiments in this study were performed at least three biological triplicates, and the data were represented as mean ± standard deviation (SD). Statistical significance was analyzed by unpaired two-tailed Student’s *t*-test, with ^∗^*P* < 0.05, ^∗∗^*P* < 0.01, and ^∗∗∗^*P* < 0.001.

## Results

### Coexistence of Multiple Types of QS Systems in NMWT1

The complete genome of *S. cyaneogriseus* ssp. *noncyanogenus* NMWT1 was sequenced using a whole-genome shotgun strategy by our group in 2015 (Wang et al., 2015). Protein BLAST analysis of the NMWT1 genome revealed the presence of three TetR family transcriptional regulatory genes, namely *scyR1* (*TU94_00975*), *scyR2* (*TU94_03165*), and *scyR3* (*TU94_11460*), which encode homologs of GBL receptors (Supplementary Table 3). ScyR1 exhibited high similarity to AvaR1 from *S. avermitilis* (70% identity) and to TylP from *S. fradiae* (60% identity); ScyR2 showed sequence similarity to TylP from *S. fradiae* (33% identity) and to JadR3 from *S. venezuelae* (33% identity), and ScyR3 showed sequence similarity to TylP from *S. fradiae* (45% identity) and to ArpA from *S. griseus* (43% identity) (Supplementary Table 3). Genes encoding the autoregulator receptors and synthases are usually clustered in the same locus in the genomes of various *Streptomyces* species (Nishida et al., 2007). Therefore, we performed protein BLAST searches to determine the function of genes close to *scyR1*, *scyR2*, and *scyR3*. Interestingly, the *scyR1* proximal genes *scyA1* (*TU94_00985*) and *TU94_00970* were found to encode proteins highly similar to the *S. avermitilis* acyl-CoA oxidase Aco (63% identities) and the cytochrome P450 hydroxylase Cyp17 (70% identities), respectively (Supplementary Table 4), suggesting a role in the synthesis of an avenolide-like compound (Kitani et al., 2011). No genes involved in the synthesis of autoregulators were found in the vicinity of *scyR2*. Upstream of *scyR3*, we found a gene homolog of GBL synthase, *scyA3* (*TU94_11455*), whose product showed 38% identity to *S. griseus* AfsA, an A-factor synthase (Supplementary Table 4) (Horinouchi, 2007).

**FIGURE 1:**
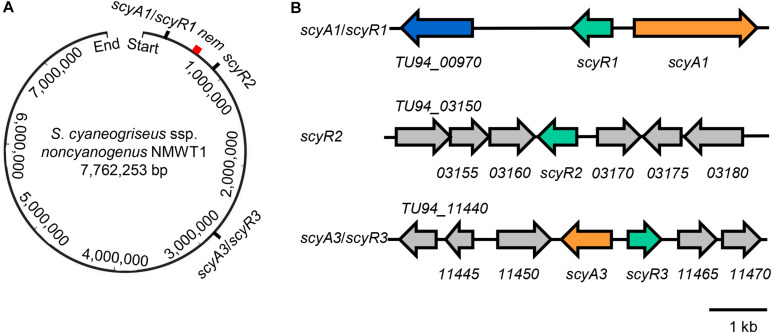
Genetic organization of *scyA1*/*scyR1*, *scyR2*, *scyA3*/*scyR3*, and *nem* cluster in the genome of *S. cyaneogriseus* ssp. *noncyanogenus* NMWT1. **(A)** Schematic representation of the relative positions of *scyA1*/*scyR1*, *scyR2*, *scyA3*/*scyR3*, and *nem* cluster on the chromosome. The red rectangle indicates *nem* cluster. **(B)** Gene organization of *scyA1*/*scyR1*, *scyR2*, *scyA3*/*scyR3* and its adjacent genes. Genes are indicated by arrows, orange: genes encoding the acyl-CoA oxidase (ScyA1) or the A-factor synthase homolog (ScyA3); green: genes encoding homologs of γ-butyrolactone (GBL) receptors; blue: the gene encoding a cytochrome P450 monooxygenase.

These findings suggest that the genome of *S. cyaneogriseus* ssp. *noncyanogenus* NMWT1 encodes two different types of QS systems and an orphan GBL receptor homolog: the *aco*/*arpA*-like system (*TU94_985*/*TU94_975*, *scyA1*/*scyR1*), the *afsA*/*arpA*-like system (*TU94_11455*/*TU94_11460*, *scyA3*/*scyR3*), and a putative *arpA* homolog (*TU94_3165*, *scyR2*) (Figure 1). The *scyA1*/*scyR1* pair is located at the left arm of the chromosome, ∼310 kb upstream of the nemadectin (*nem*) cluster (*TU94_02425*-*TU94_02495*; GenBank accession No. AB363939). *scyR2* is also located at the left arm of the chromosome but is ∼240 kb downstream of the *nem* cluster; the *scyA3*/*scyR3* locus is close to the central region of the genome, ∼2,160 kb from the *nem* cluster (Figure 1A). It should be noted that, in the subsequent gene deletion experiments, we found that the flanking DNA sequences of *scyR1* and the ORF of *scyA1* differed from those obtained from the published genome sequence (Wang et al., 2015). Therefore, we corrected this region by PCR amplification and DNA sequencing. After careful analysis, we re-annotated the coding sequence of *scyA1* and found that *TU94_00980* did not exist (Figure 1B). The corrected nucleotide sequence was submitted to the GenBank database (accession No. MT563325).

### Transcriptional Profiles of *scyA1/scyR1*, *scyR2*, and *scyA3/scyR3*

**FIGURE 2:**
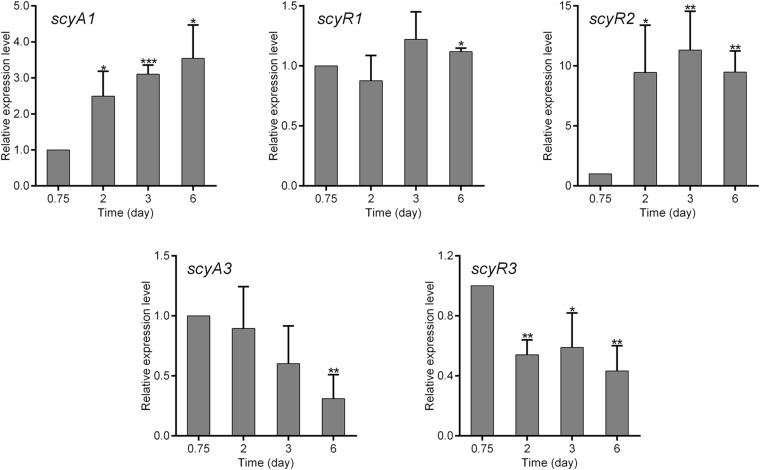
Quantitative real-time RT-PCR (qRT-PCR) analysis of the genes associated with the quorum-sensing systems in *S. cyaneogriseus* ssp. *noncyanogenus* NMWT1. The transcriptional levels of *scyA1*, *scyR1*, *scyR2*, *scyA3*, and *scyR3* are presented relative to that of NMWT1 sample collected after fermentation for 0.75 day, which was arbitrarily assigned a value of 1. 16S rRNA transcription was monitored and used as the internal control. Data are presented as the averages of three independent experiments conducted in triplicate. Error bars show standard deviations. *P*-values were determined by Student’s *t*-test. *P < 0.05, **P < 0.01, and ***P < 0.001.

To investigate the functions of *scyA1*/*scyR1*, *scyR2* and *scyA3*/*scyR3*, we analyzed their transcriptional profiles during nemadectin production. Total RNAs were isolated from the mycelia of NMWT1 after cultivation for various days (0.75, 2, 3, and 6 days) in nemadectin fermentation medium, and the transcriptional levels of *scyAs* and *scyRs* were determined by quantitative real-time RT-PCR (qRT-PCR). Transcription of *scyA1* increased gradually, whereas that of *scyR1* remained constant (Figure 2). The transcriptional level of *scyR2* was very low at 0.75 day, but showed a sharp increase from 0.75 day onward. *scyA3* and *scyR3* exhibited similar transcriptional profiles; their levels were highest on 0.75 day and then decreased, although still at relatively high levels. These results indicate that although all five genes were expressed, their transcriptional patterns differed, suggesting differential regulatory roles in *S. cyaneogriseus*.

### Roles of *scyA1*, *scyR1*, *scyR2*, *scyA3*, and *scyR3* in Morphological Development and Nemadectin Production

To determine the *in vivo* functions of *scyA1*, *scyR1*, *scyR2*, *scyA3*, and *scyR3*, we constructed deletion mutants using CRISPR/Cas9-mediated genome-editing (Huang et al., 2015). The *scyA1* mutant (ΔscyA1) had a 1,239-bp deletion in the *scyA1* ORF, ΔscyR2 a 333-bp deletion in the *scyR2* ORF, ΔscyA3 a 612-bp deletion in the *scyA3* ORF, and ΔscyR3 a 633-bp deletion in the *scyR3* ORF. The presence of these deletions was confirmed by PCR (Supplementary Figure 1) and DNA sequencing (data not shown). However, our efforts to construct a *scyR1* deletion mutant (ΔscyR1) failed. Hence, to assess the function of *scyR1*, we employed the dCas9-based CRISPR interference (CRISPRi) system to repress *scyR1* transcription elongation. The *scyR1* CRISPRi plasmid was constructed as described previously (Supplementary Figure 2A) (Zhao et al., 2018) and was used to generate the *scyR1* repression strain RscyR1. The *scyR1* transcript level was assessed by qRT-PCR, which confirmed that the CRISPRi-mediated *scyR1* silencing was successful (Supplementary Figure 2B).

**FIGURE 3:**
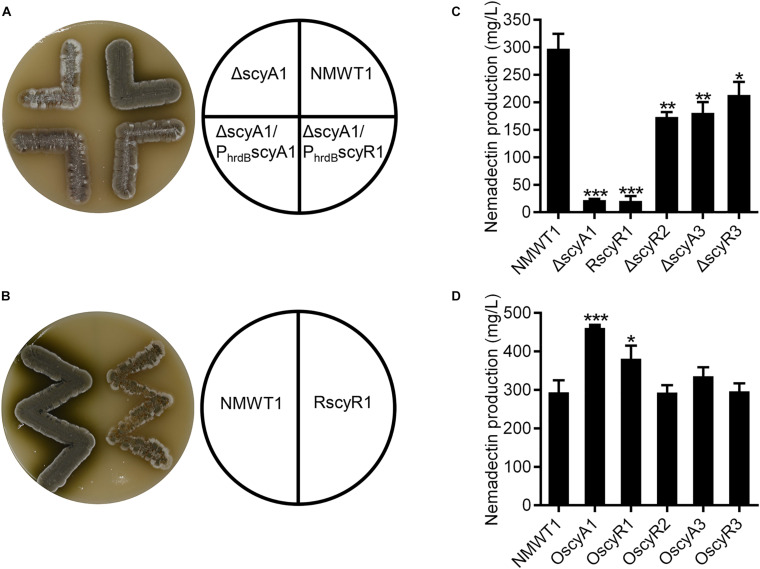
Effects of *scyA1*, *scyR1*, *scyR2*, *scyA3*, and *scyR3* on sporulation and nemadectin production. **(A)** Morphological differentiation among strains NMWT1, ΔscyA1, ΔscyA1/P_hrdB_scyA1, and ΔscyA1/P_hrdB_scyR1 grown on ISP3 agar medium at 37°C for 4 days. **(B)** Morphological differentiation among strains NMWT1 and RscyR1 grown on ISP3 agar medium at 37°C for 4 days. **(C)** Effects of these five genes’ deletion or inhibition on nemadectin production. Error bars show standard deviations. *P < 0.05, **P < 0.01, and ***P < 0.001. **(D)** Effects of separate overexpression of the five genes on nemadectin production.

To assess the phenotypes of the ΔscyA1, RscyR1, ΔscyR2, ΔscyA3, and ΔscyR3 strains, we cultured them on ISP3 agar medium at 37°C for 4 days. Compared with NMWT1, ΔscyA1 grew slowly, produced small amounts of spores, and did not produce diffusible dark-olive pigment (Figure 3A). RscyR1 also showed impaired growth and spore production and formed colonies with clear-white edges (Figure 3B). To clarify whether the phenotype of ΔscyA1 was due to *scyA1* deletion, an integrative plasmid pSET152 containing *scyA1* ORF downstream of the constitutive *hrdB* promoter was introduced into ΔscyA1, generating the complementation strain ΔscyA1/P_hrdB_scyA1. ΔscyA1/P_hrdB_ scyA1 restored spore formation, although the spore amounts were less than those of the parental strain NMWT1 (Figure 3A). ΔscyR2, ΔscyA3, and ΔscyR3 showed no distinct alterations (data not shown). These results suggest that *scyA1* and *scyR1* play important roles in the morphological development of *S. cyaneogriseus*, in contrast to *scyR2*, *scyA3*, and *scyR3*.

Compared with NMWT1, all the mutant strains exhibited decreased nemadectin production. Particularly, nemadectin production was decreased by 92, 93, 42, 39, and 28% in ΔscyA1, RscyR1, ΔscyR2, ΔscyA3, and ΔscyR3, respectively (Figure 3C). These data suggest that all five genes play positive roles in nemadectin production, with *scyA1* and *scyR1* being the most important. To determine the effects of overexpression of the five genes on nemadectin production, we introduced pSET152-P_hrdB_scyA1, pSET152-P_hrdB_scyR1, pSET152-P_hrdB_scyR2, pSET152-P_hrdB_scyA3 or pSET152-P_hrdB_scyR3 (here, every gene was controlled by the *hrdB* promoter) into NMWT1 to obtain OscyA1, OscyR1, OscyR2, OscyA3, and OscyR3 strains, respectively. Nemadectin production was enhanced by 56% in OscyA1 and by 29% in OscyR1; however, no significant changes in nemadectin production were observed in OscyR2, OscyA3, and OscyR3 (Figure 3D).

### ScyA1, ScyR1, ScyR2, and ScyR3 Activate the Transcription of *nemR* and *nemA1-2*

**FIGURE 4:**
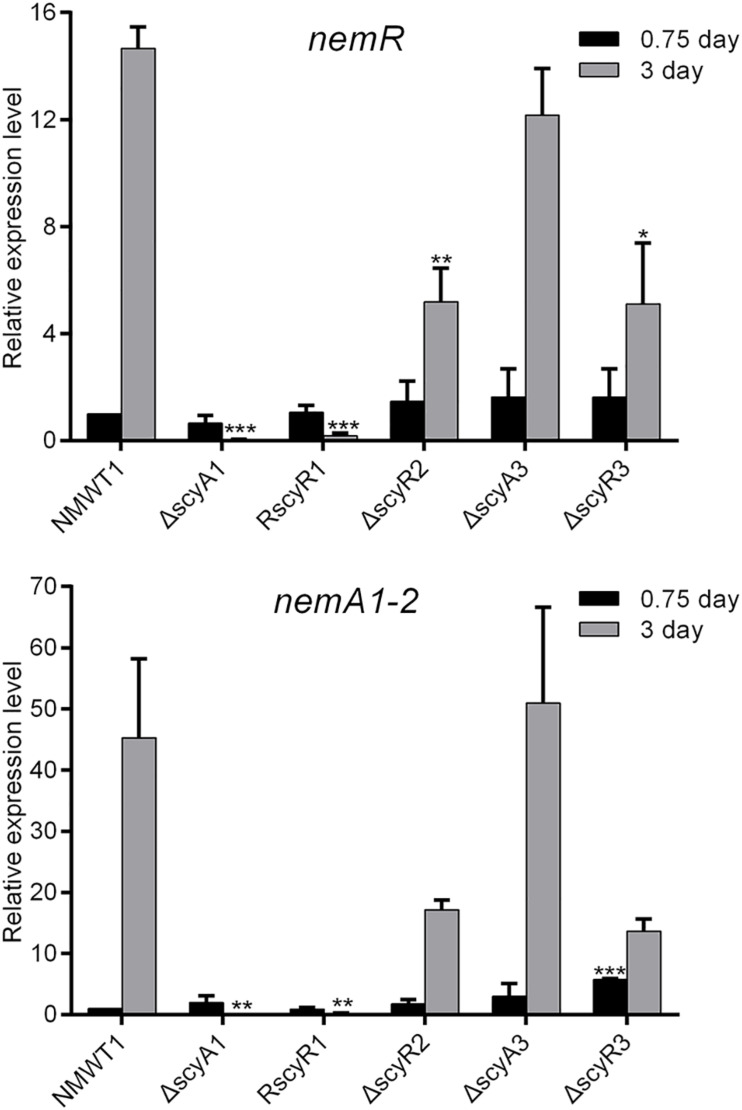
Transcriptional analyses of *nemR* and *nemA1-2* in NMWT1, ΔscyA1, RscyR1, ΔscyR2, ΔscyA3, and ΔscyR3 strains. The transcriptional levels of *nemR* and *nemA1-2* in NMWT1 sample collected after fermentation for 0.75 day were assigned a value of 1. 16S rRNA transcription was monitored and used as the internal control. Data are presented as the averages of three independent experiments conducted in triplicate. *P < 0.05, **P < 0.01, and ***P < 0.001.

Next, we investigate the roles of *scyA1*, *scyR1*, *scyR2*, *scyA3*, and *scyR3* in the expression of the nemadectin biosynthesis-related genes *nemR* (cluster-situated activator gene) and *nemA1-2* (type I polyketide synthase gene) by qRT-PCR. Little to no *nemR* transcription was observed in ΔscyA1 and RscyR1; the *nemR* transcription level was significantly lower in ΔscyR2 and ΔscyR3 than in NMWT1 (Figure 4). Similar results were obtained for *nemA1-2* (Figure 4). In contrast, *nemR* and *nemA1-2* transcriptional levels in ΔscyA3 were similar to those in NMWT1 (Figure 4). These results suggest that ScyA1, ScyR1, ScyR2, and ScyR3 control nemadectin production by activating the transcription of *nem* genes.

To determine whether ScyR1, ScyR2, and ScyR3 activate the transcription of *nem* genes directly, EMSAs were performed. The His_6_-tagged ScyR1 and ScyR3 and GST-tagged ScyR2 were expressed in *E. coli* (Supplementary Figure 3). The promoter regions of *nem* cluster genes (*nemR*, *nemA1-1/A1-2/A2*, *nemC*, and *nemA4/A3/E/D*) were used as probes, and the *hrdB* promoter was used as a negative control. None of the three regulatory proteins could bind to any of the *nem* promoter probes (data not shown), indicating that they indirectly activate the expression of *nem* genes.

### ScyA1 Promoted Morphological Differentiation and Nemadectin Production by Activating *scyR1* Transcription

**FIGURE 5:**
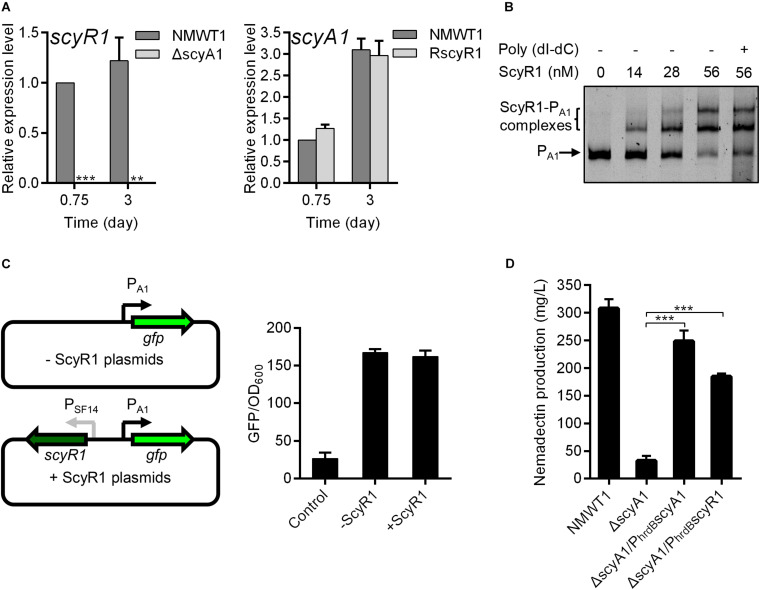
The regulatory relationships between *scyA1* and *scyR1*. **(A)** qRT-PCR analysis of *scyA1* and *scyR1* in NMWT1, ΔscyA1 and RscyR1 strains. *P < 0.05, **P < 0.01, and ***P < 0.001. **(B)** EMSA of ScyR1 binding to the promoter region P_A1_. Each lane contains 10 ng of DNA probes. Line 6 contains 100-fold non-specific poly(dI-dC). The promoter region of *scyA1* was 775-bp. DNA-protein complexes are indicated by brackets. Free probes are indicated by arrows. **(C)** An illustration of the reporter plasmids that are used for identification of regulatory effects of ScyR1 on the promoter P_A1_ in *E. coli*. All values are in relative fluorescence unit (GFP/OD_600_) and represent the averages of at least three independent readings. **(D)** HPLC analysis of nemadectin production in strains NMWT1, ΔscyA1, ΔscyA1/P_hrdB_scyA1, and ΔscyA1/P_hrdB_scyR1 cultured in fermentation medium for 9 days.

The changes in the colony phenotype and nemadectin production were more remarkable in ΔscyA1 and RscyR1; hence, we further investigated the regulatory role of the ScyA1/ScyR1 system. First, we determined the effects of *scyA1* and *scyR1* on each other’s expression by qRT-PCR. *scyR1* transcripts were abolished in ΔscyA1, indicating that ScyA1 is essential for *scyR1* transcription. The *scyA1* expression level in RscyR1 was comparable with that in NMWT1 (Figure 5A), suggesting that ScyR1 does not affect *scyA1* expression. To further determine the regulatory relationship between ScyR1 and the *scyA1* promoter (P_A1_, also known as the intergenic region between *scyA1* and *scyR1*), EMSAs and the green fluorescent protein (GFP) reporter experiment in *E. coli* were performed. EMSAs showed that the ScyR1–P_A1_ complexes formed as the amount of ScyR1 increased, indicating the direct regulation of ScyR1 toward P_A1_ (Figure 5B and Supplementary Figure 4). However, the fluorescence intensity in strain containing pSET152:P_A1_*gfp*:SF14*scyR1* was similar to that in strain harboring pSET152:P_A1_*gfp* (Figure 5C). This finding corroborated the qRT-PCR results but contradicted the EMSA findings. The regulation of a neighboring oppositely transcribed gene has been recognized as a general feature of TetR-like regulatory proteins (Zhang et al., 2013), but in this work, we found that ScyR1 did not regulate *scyA1* expression.

The results described above clearly showed that ScyA1 is an upstream regulator of *scyR1*. This promoted us to speculate that ScyA1 may function by controlling *scyR1* transcription. To verify our speculation, we introduced an integrative plasmid containing a single copy of *scyR1* under the control of the *hrdB* promoter into ΔscyA1. The resulted strain was named as ΔscyA1/P_hrdB_scyR1, in which the *scyR1* transcript level exhibited a 4.4-fold increase compared with that in NMWT1 (Supplementary Figure 5). ΔscyA1/P_hrdB_scyR1 colonies were similar to those formed by the *scyA1* complementation strain (ΔscyA1/P_hrdB_scyA1), suggesting that ScyA1 promotes morphological differentiation in a ScyR1-dependent manner (Figure 3A). Nemadectin production was also partly restored, reaching 74% relative to that in ΔscyA1/P_hrdB_scyA1 and 60% relative to that in NMWT1, indicating that ScyA1 positively regulates nemadectin production in a ScyR1-dependent manner (Figure 5D). The transcript levels of *nemR* were also significantly increased in ΔscyA1/P_hrdB_scyR1 (59% relative to NMWT1; Supplementary Figure 6). These data suggest that ScyA1 positively regulates morphological differentiation and nemadectin production by activating *scyR1* transcription.

### *scyR1* Silencing in NMWT1 Activates the Expression of Numerous Metabolite Biosynthetic Core Genes

**FIGURE 6:**
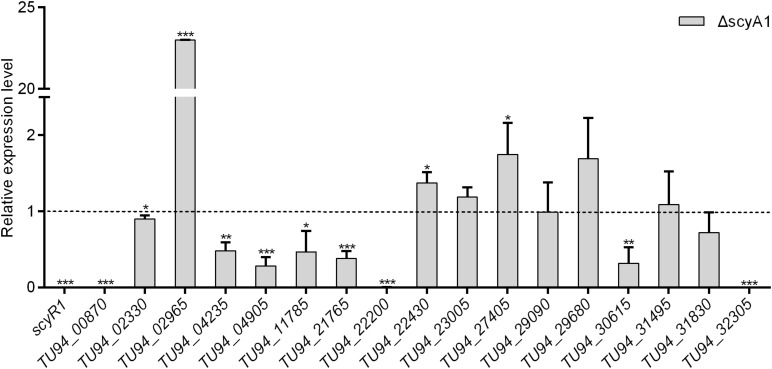
Transcriptional analysis of the secondary metabolite biosynthetic core genes in NMWT1 and ΔscyA1. All RNA samples were isolated from 3 days cultures. The transcript level of each biosynthetic core gene in NMWT1 sample collected after fermentation for 3 days was assigned a value of 1 (represented by the dotted line). Data are presented as the averages of three independent experiments conducted in triplicate. *P < 0.05, **P < 0.01, ***P < 0.001.

**FIGURE 7:**
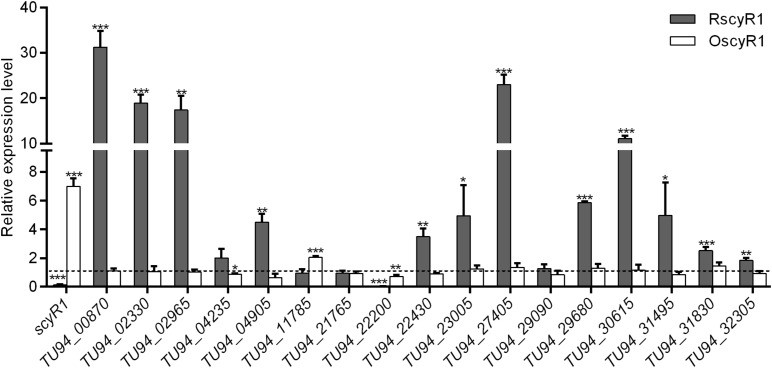
qRT-PCR transcriptional analysis of secondary metabolite biosynthetic core genes in NMWT1, RscyR1, and OscyR1. All RNA samples were isolated from 3 days cultures. The transcriptional level of each core gene in NMWT1 collected after fermentation for 3 days was assigned a value of 1 (represented by the dotted line). *P < 0.05, **P < 0.01, and ***P < 0.001.

To assess the function of ScyA1/ScyR1 in secondary metabolism, we investigated the transcript levels of putative secondary metabolite biosynthetic gene clusters within the *S. cyaneogriseus* genome. Seventeen clusters (including PKS-type, NRPS-type, PKS-NRPS-type, terpene, melanin, and bacteriocin) identified by the antiSMASH software were selected, as they exhibited high similarities with well-known secondary metabolite gene clusters, and their corresponding chemical backbones can be predicted (Supplementary Table 5). The transcript levels of *scyR1* and 17 representative biosynthetic core genes from the biosynthetic gene clusters were measured by qRT-PCR in strains NMWT1, ΔscyA1 and RscyR1. The transcript levels of *TU94_00870*, *TU94_02330*, *TU94_04235*, *TU94_04905*, *TU94_11785*, *TU94_21765*, *TU94_22200*, *TU94_30615*, and *TU94_32305* were significantly lower in ΔscyA1 than in NMWT1, whereas that of *TU94_02965* was higher (Figure 6). These findings suggest that ScyA1 predominantly functions as an activator rather than as a repressor. Interestingly, the transcriptional levels of 12 genes (e.g., *TU94_00870*, *TU94_02330*, *TU94_02965*, *TU94_04905*, *TU94_22430*, *TU94_23005*, *TU94_27405*, *TU94_29680*, *TU94_30615*, *TU94_31495*, *TU94_31830*, and *TU94_32305*) were significantly higher in RscyR1 than in NMWT1, revealing that ScyR1 represses the expression of these biosynthetic genes (Figure 7). To further confirm the repressive role of ScyR1, we measured the expression levels of *scyR1* and the 17 biosynthetic core genes in OscyR1 strain. *scyR1* transcript level exhibited a 6.9-fold increase compared with that in NMWT1 (Figure 7). As expected, the transcriptional levels of the 12 genes were similar to or slightly lower than those in NMWT1 (Figure 7), suggesting that ScyR1 was indeed a repressor and genetic manipulation of *scyR1* was effective in activating cryptic secondary metabolite biosynthetic genes.

### Culture Extracts From NMWT1 Could Promote *scyR1* Transcription, Nemadectin Production, and Modulate the Binding Activity of ScyR1

**FIGURE 8:**
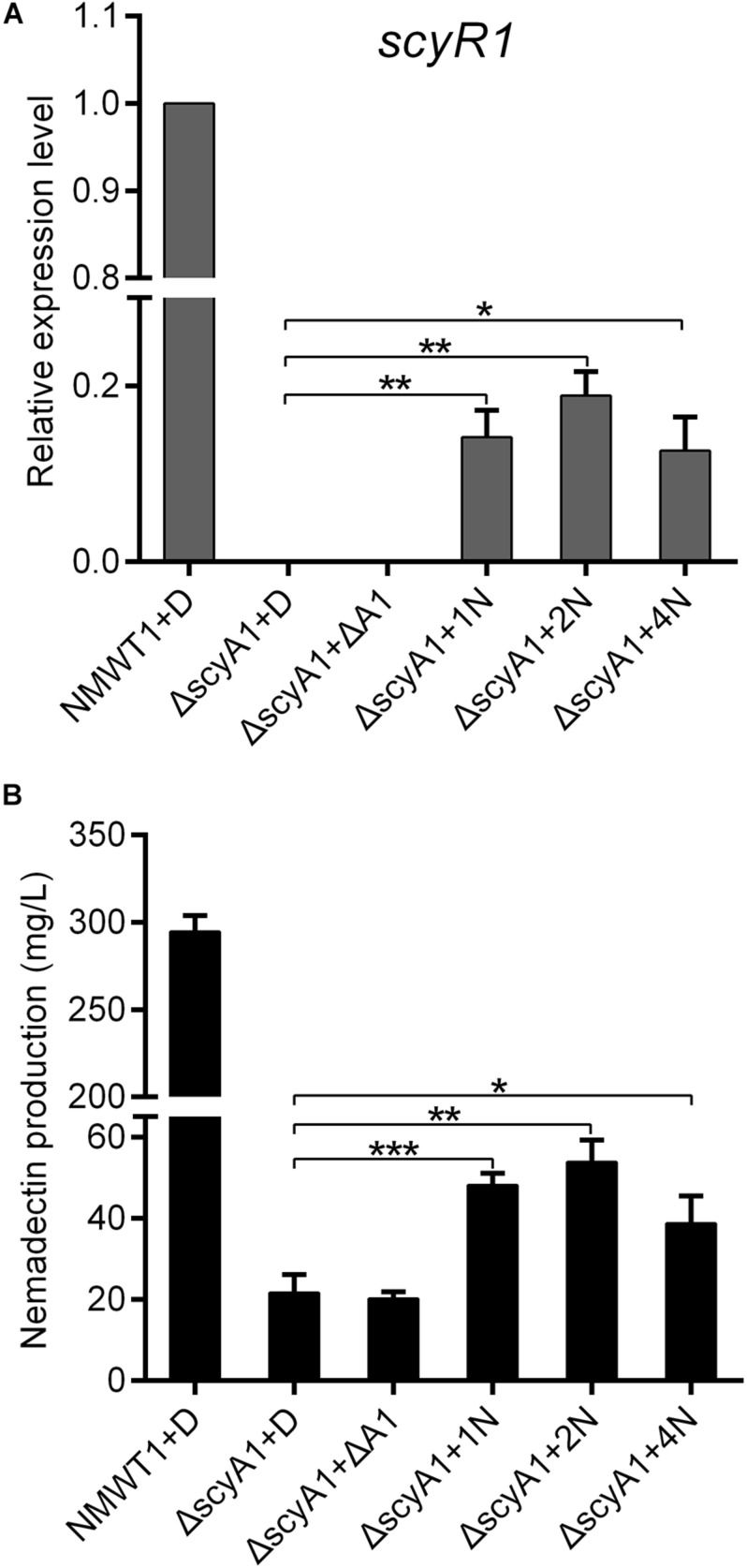
Effect of exogenously added culture extracts of NMWT1 or ΔscyA1 on *scyR1* expression and nemadectin production. **(A)** qRT-PCR transcription profile analysis of *scyR1*. Total RNAs were isolated from these six samples after fermentation for 3 days. **(B)** Nemadectin production in the *scyA1* mutant with the ethyl acetate extracts from cultures of NMWT1 or ΔscyA1. + D: DMSO was added to 30 mL fermentation medium; + ΔA1: ethyl acetate extracts from 30 mL ΔscyA1 seed cultures broth were added to 30 mL ΔscyA1 fermentation medium; + 1/2/4N: ethyl acetate extracts from 30/60/120 mL seed culture broths of NMWT1 were added separately to 30 mL ΔscyA1 fermentation medium. All additions were equal in volume. *P < 0.05, **P < 0.01, and ***P < 0.001.

ScyA1 and ScyR1 constitute an Aco/ArpA-like QS system, in which ScyA1 is the key autoregulator synthase. It is possible that ScyA1 may activate expression of target genes by producing autoregulators. To test this possibility, culture extracts from NMWT1 and ΔscyA1 were prepared and added separately into ΔscyA1 cultures. As shown in Figure 8A, transcripts of *scyR1* were abolished in ΔscyA1, but was increased significantly after the addition of NMWT1 culture extracts, although at a level relatively lower than those of NMWT1, indicating that the autoregulator determined by ScyA1 could activate the expression of *scyR1*. As expected, culture extracts from NMWT1 promoted nemadectin production in ΔscyA1, but culture extracts from ΔscyA1 were unable to elicit nemadectin (Figure 8B), indicating that the autoregulators synthesized by ScyA1 could trigger nemadectin biosynthesis. To determine whether ScyR1 is the receptor of autoregulators synthesized by ScyA1, culture extracts of NMWT1 and ΔscyA1 were also assayed for their influence on the binding activity of ScyR1 to P_A1_. As expected, culture extracts from NMWT1 could dissociate ScyR1 from P_A1_ whereas the extracts from ΔscyA1 could not inhibit the formation of ScyR1-P_A1_ complexes (Supplementary Figure 7). This indicated that ScyR1 may be the receptor of autoregulators determined by ScyA1. These preliminary results suggest the potential of ScyA1 as a QS signal synthase and ScyR1 as an autoregulator receptor. The structure of the autoregulator produced by ScyA1 needs to be determined in the future, which will help to understand the ScyA1/ScyR1 regulatory process well.

## Discussion

Quorum-sensing systems are important for secondary metabolism and morphological development in *Streptomyces* (Kitani et al., 2011). Although substantial progress has been made in understanding the mechanisms by which ArpA homolog regulates antibiotic biosynthesis (Liu et al., 2013), the pathways involved in the autoregulator synthase-mediated initiation of antibiotic production are poorly understood. Here, we used the nemadectin-producing strain *Streptomyces cyaneogriseus* ssp. *noncyanogenus* NMWT1 and found for the first time that the Aco/ArpA-like system ScyA1/ScyR1 is required for nemadectin production. Specifically, ScyA1 induces *scyR1* transcription, and in turn ScyR1 promotes nemadectin production. Moreover, the substantial increase in transcripts of most of the metabolite biosynthetic core genes after *scyR1* silencing reveals a method to activate the expression of silent biosynthetic gene clusters.

The regulatory relationship between the autoregulator synthase (AfsA or Aco-like) and the cognate ArpA receptor in *Streptomyces*, and how the two components coordinately modulate secondary metabolism, have gained increasing attention over the last years. In this study, we identified that one Aco/ArpA-like system, ScyA1/ScyR1, is involved in nemadectin production and morphological development in *Streptomyces cyaneogriseus* ssp. *noncyanogenus* NMWT1. ScyA1 and ScyR1 are homologs of the *S. avermitilis* proteins Aco and AvaR1, respectively; however, the regulatory relationship between ScyA1 and ScyR1 and their regulatory pathway for antibiotic production differ from those of Aco and AvaR1. In *S. avermitilis*, Aco is required for avermectin biosynthesis; AvaR1 directly represses *aco* expression and can also directly inhibiting avermectin production by repressing transcription of the cluster-situated activator gene *aveR* (Zhu et al., 2017). For the ScyA1/ScyR1 system, both ScyA1 and ScyR1 have positive effects on nemadectin production; although ScyR1 does not affect the expression of *scyA1*, ScyA1 is essential for *scyR1* expression. Interestingly, constitutive expression of *scyR1* in the *scyA1* mutant led to restoration of nemadectin production to levels close to those in the *scyA1* complementation strain ΔscyA1/P_hrdB_scyA1 and the wild-type NMWT1, revealing that the autoregulator synthase controls nemadectin production by activating the expression of the cognate receptor. The AfsA/ArpA-like system components JadW1 (GBL synthase) and JadR3 from *Streptomyces venezuelae* have similar antibiotic production phenotypes to those of ScyA1 and ScyR1, respectively (Zou et al., 2014). Both *jadW1* and *jadR3* positively regulate jadomycin production, and JadW1 is required for *jadR3* expression. However, unlike ScyA1/ScyR1, JadR3 directly represses the expression of *jadW1* and promotes jadomycin production by activating the expression of *jadR1*; the mechanism by which JadW1 triggers jadomycin production is unclear (Zou et al., 2014). Other QS systems including ScbA/ScbR, BarX/BarA, FarX/FarA, AfsA/ArpA, SabA/SabR1, and SbbA/SbbR, have also been reported to control antibiotic biosynthesis via complex regulatory networks (Waki et al., 1997; Kawachi et al., 2000; Takano et al., 2001; He et al., 2018). Therefore, different QS systems may regulate antibiotic biosynthesis via different pathways. Here, we found that ScyA1 promotes nemadectin production by activating the expression of *scyR1*, representing a previously unknown QS regulatory pathway. It should be noted that, the activation of *scyR1* by ScyA1 may not be achieved by eliminating the inhibition of ScyR2 or ScyR3, because transcription of *scyR1* in ΔscyR2 and ΔscyR3 decreased, indicating the positive role of ScyR2 and ScyR3 toward *scyR1* expression (our unpublished data). Therefore, the mechanism by which ScyA1 activates the expression of *scyR1* is somewhat unclear. Additionally, future studies are needed to identify the mechanisms by which ScyR1 indirectly regulates the biosynthesis of nemadectin.

ScyA1 promotes nemadectin production by activating the transcription of *scyR1*; in turn, ScyR1 is required for the expression of the *nem* cluster. However, *scyR1* overexpression in the *scyA1* mutant did not increase *nemR* expression or nemadectin production to levels higher than those in NMWT1, despite that the *scyR1* expression level was higher than that in NMWT1 (Supplementary Figure 5). Similarly, although the *scyR1* expression level was markedly higher in OscyR1 than NMWT1 (Figure 7), but nemadectin production was only 29% higher in OscyR1 than in NMWT1 (Figure 3D). Therefore, we believe that the role of ScyR1 in nemadectin production is complex. In addition to regulating nemadectin production, ScyR1 also regulates sporulation. Hence, ScyR1 is a pleiotropic regulator required for both secondary metabolism and morphological development. It is possible that the transition from aerial mycelium to mature spores is accompanied by complex ScyR1-dependent or -independent physiological changes (Zhou et al., 2020), which may also be closely linked to nemadectin production. Thus, ScyR1 may affect nemadectin production via various interconnected regulatory pathways. Future comprehensive studies aiming to identify ScyR1 target genes involved in nemadectin production are required to improve the yield of nemadectin biosynthesis.

## Data Availability Statement

The original contributions presented in the study are included in the article/Supplementary Material, further inquiries can be directed to the corresponding authors.

## Author Contributions

YZ, WX, and HL designed the research and wrote the manuscript. HL performed the experiments. SL, JW, and XW helped modify the article. All authors contributed to the article and approved the submitted version.

## Conflict of Interest

The authors declare that the research was conducted in the absence of any commercial or financial relationships that could be construed as a potential conflict of interest.

## Supplementary Material

Supplementary Figure 1 Confirmation of *scyA1*, *scyR2*, *scyA3*, and *scyR3* deletion by PCR amplification.

Supplementary Figure 2 Confirmation of *scyR1* repression by qRT-PCR.

Supplementary Figure 3 SDS-PAGE analysis of the purified ScyR1-His_6_ (26.0 kDa), ScyR2-GST (51.8 kDa), and ScyR3-His_6_ (24.1 kDa).

Supplementary Figure 4 EMSA of ScyR1 binding to the promoter P_hrdB_.

Supplementary Figure 5 qRT-PCR analysis of *scyR1* in *S. cyaneogriseus* ssp. *noncyanogenus* NMWT1, ΔscyA1 and ΔscyA1/P_hrdB_scyR1.

Supplementary Figure 6 qRT-PCR analysis of *nemR* in *S. cyaneogriseus* ssp. *noncyanogenus* NMWT1, ΔscyA1 and ΔscyA1/P_hrdB_scyR1.

Supplementary Figure 7 Influences of ethyl acetate extracts from NMWT1 and ΔscyA1 on the DNA binding activity of ScyR1.

Supplementary Table 1 Strains and plasmids used in this study.

Supplementary Table 2 Primers used in this study.

Supplementary Table 3 BlastP analysis of ScyR1, ScyR2, ScyR3 and their homologs^∗∗^.

Supplementary Table 4 BlastP analysis of ScyA1, TU94_00970, ScyA3 and their homologs.

Supplementary Table 5 17 secondary metabolite biosynthetic core genes and the corresponding associated biosynthetic gene clusters in *S. cyaneogriseus* ssp. *noncyanogenus* NMWT1.
