# Supplementary material for: Elucidation of the Activation Pathways of ScyA1/ScyR1, an Aco/ArpA-Like System That Regulates the Expression of Nemadectin and Other Secondary Metabolic Biosynthetic Genes
Source: Front Bioeng Biotechnol. 2020 Nov 3;8:589730. doi: 10.3389/fbioe.2020.589730 (PMC7670052; doi:10.3389/fbioe.2020.589730)
Supplement: Supplementary Figure 1 — Confirmation of scyA1, scyR2, scyA3, and scyR3 deletion by PCR amplification. [file Data_Sheet_1.pdf]

Supplementary Material

1 Supplementary Figures

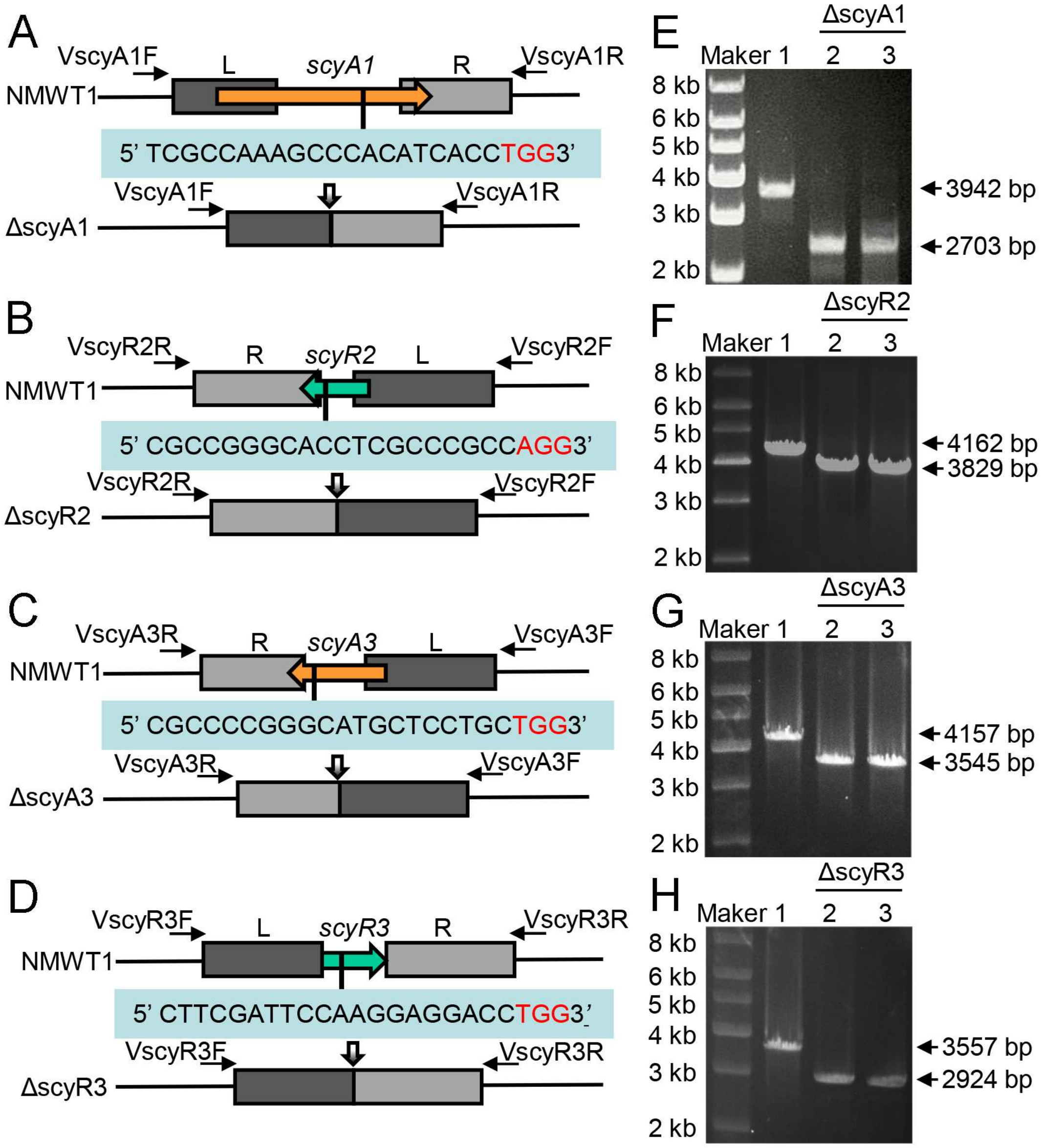

Supplementary Figure 1. Confirmation of *scyA1*, *scyR2*, *scyA3* and *scyR3* deletion by PCR amplification. (**A**-**D**) Diagrams of *scyA1*, *scyR2*, *scyA3* and *scyR3* disruption constructions. Each verification primer is designed outside of the upstream (L) or downstream (R) sequence that used for constructing disruption mutants. 20-nt protospacer sequences of crRNAs are annotated in the diagrams. (**E**-**H**) Agarose gel electrophoresis showing PCR amplified fragments. Line 1: the PCR template was the genomic DNA from *S. cyaneogriseu*s ssp. *noncyanogenus* NMWT1; Lane 2 and 3: the PCR templates were the genomic DNAs from two independent mutants as indicated, respectively.

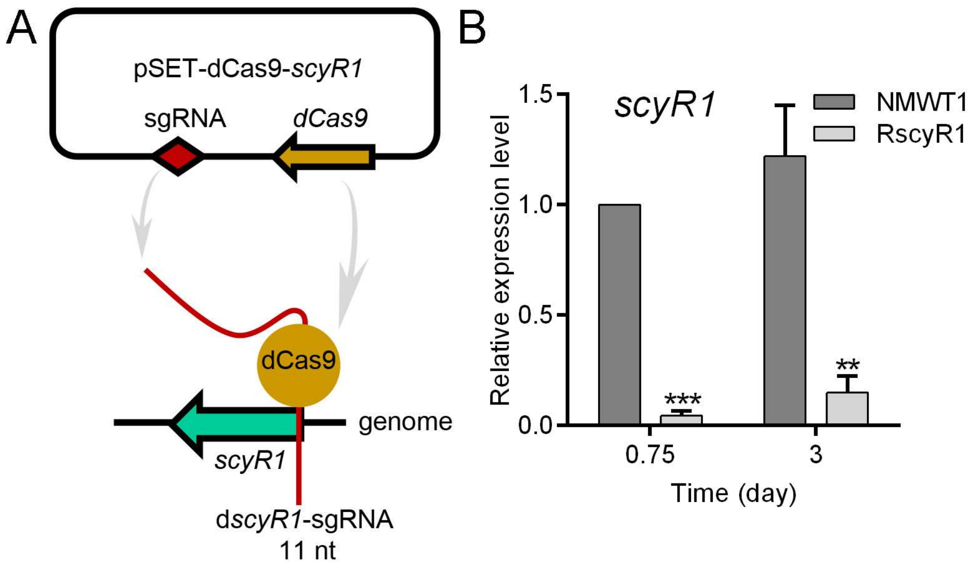

Supplementary Figure 2. Confirmation of *scyR1* repression by qRT-PCR. (**A**) Diagram of the CRISPR/dCas9-mediated *scyR1* repression. (**B**) Transcriptional analysis of *scyR1* in *S. cyaneogriseu*s ssp. *noncyanogenus* NMWT1 and RscyR1 by qRT-PCR to check the repression efficiency. The transcriptional level of *scyR1* in NMWT1 sample collected after fermentation for 0.75 day was arbitrarily assigned a value of 1. 16S rRNA transcription was monitored and used as the internal control. Data are presented as the averages of three independent experiments conducted in triplicate. Error bars show standard deviations. *P*-values were determined by Student's t-test. **, $P < 0.01$. ***, $P < 0.001$.

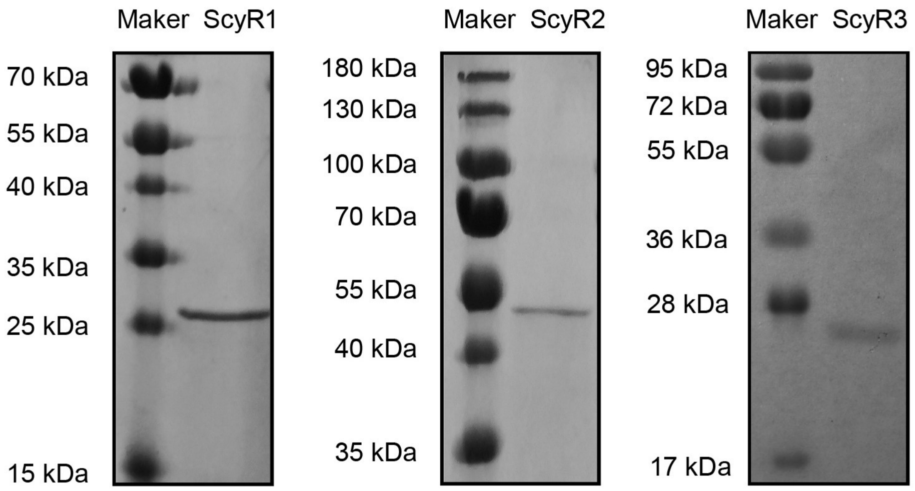

Supplementary Figure 3. SDS-PAGE analysis of the purified ScyR1-His$_6$ (26.0 kDa), ScyR2-GST (51.8 kDa) and ScyR3-His$_6$ (24.1 kDa).

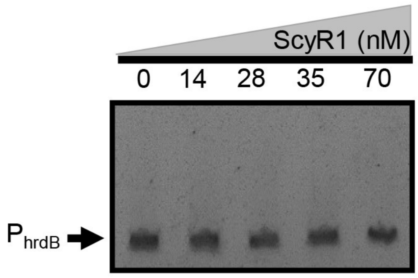

Supplementary Figure 4. EMSA of ScyR1 binding to the promoter P_{hrdB}. P_{hrdB}: the promoter region of *hrdB*. Each lane contains 10 ng of DNA probes. The promoter region of *hrdB* was prepared as 443-bp probes. Free probes are indicated by arrows.

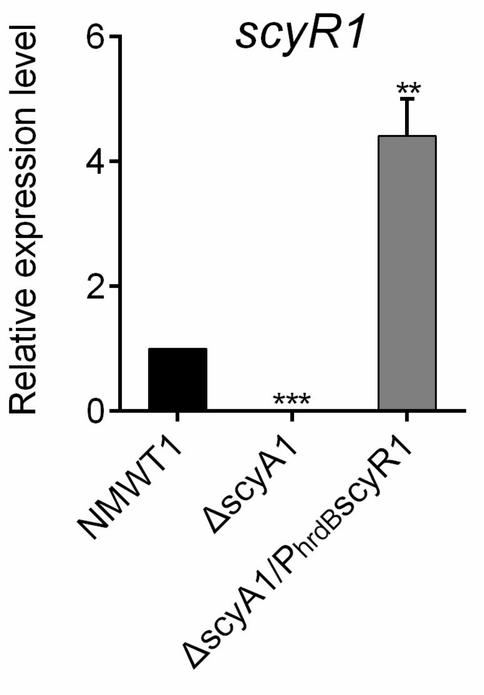

Supplementary Figure 5. qRT-PCR analysis of *scyR1* in *S. cyaneogriseus* ssp. *noncyanogenus* NMWT1, ΔscyA1 and ΔscyA1/P_{hrdB}scyR1. Error bars show standard deviations. *P*-values were determined by Student's t-test. **, $P < 0.01$. ***, $P < 0.001$.

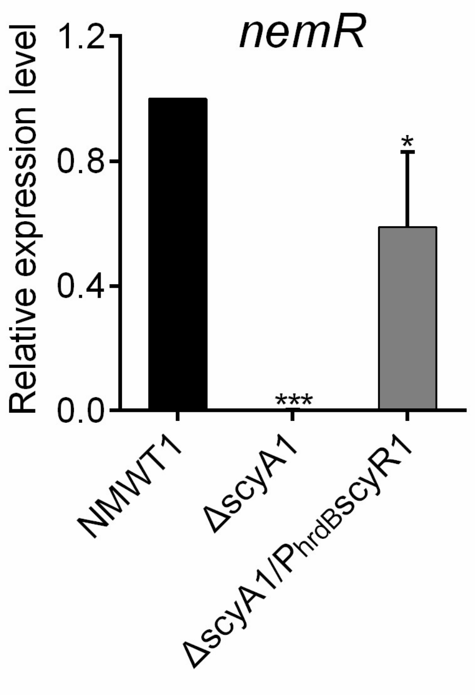

Supplementary Figure 6. qRT-PCR analysis of *nemR* in *S. cyaneogriseus* ssp. *noncyanogenus* NMWT1, ΔscyA1 and ΔscyA1/P_{hrdB}scyR1. Error bars show standard deviations. *P*-values were determined by Student's t-test. *, $P < 0.05$. ***, $P < 0.001$.

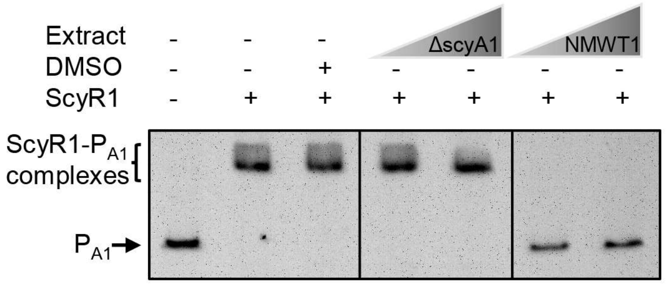

Supplementary Figure 7 Influences of ethyl acetate extracts from NMWT1 and ΔscyA1 on DNA binding activity of ScyR1. EMSAs of ScyR1 (70 nM) on probe P_{A1} were performed with culture extracts in a series of two-fold dilution steps. DMSO was used as a solvent control.
