# Supplementary material for: Elucidation of the Activation Pathways of ScyA1/ScyR1, an Aco/ArpA-Like System That Regulates the Expression of Nemadectin and Other Secondary Metabolic Biosynthetic Genes
Source: Front Bioeng Biotechnol. 2020 Nov 3;8:589730. doi: 10.3389/fbioe.2020.589730 (PMC7670052; doi:10.3389/fbioe.2020.589730)
Supplement: Supplementary Table 1 — Strains and plasmids used in this study. [file Table_1.docx]

# *Supplementary Material*

1. **Supplementary Tables**

## Supplementary Table 1. Strains and plasmids used in this study.

| Strain or plasmid | Description | Source or reference |
| --- | --- | --- |
| *S. cyaneogriseus* ssp. *noncyanogenus* | | |
| NMWT1 | Wild-type producer for nemadectin | Laboratory stock |
| ΔscyA1 | *scyA1* deletion mutant | This study |
| OscyA1 | NMWT1 containing pSET152-P_hrdB_scyA1 | This study |
| RscyR1 | *scyR1* repression mutant | This study |
| OscyR1 | NMWT1 containing pSET152-P_hrdB_scyR1 | This study |
| ΔscyR2 | *scyR2* deletion mutant | This study |
| OscyR2 | NMWT1 containing pSET152-P_hrdB_scyR2 | This study |
| ΔscyA3 | *scyA3* deletion mutant | This study |
| OscyA3 | NMWT1 containing pSET152-P_hrdB_scyA3 | This study |
| ΔscyR3 | *scyR3* deletion mutant | This study |
| OscyR3 | NMWT1 containing pSET152-P_hrdB_scyR3 | This study |
| ΔscyA1P_hrdB_scyA1 | ΔscyA1 containing pSET152-P_hrdB_scyA1 | This study |
| ΔscyA1/P_hrdB_scyR1 | ΔscyA1 containing pSET152-P_hrdB_scyR1 | This study |
| *E. coli* strains | | |
| JM109 | Cloning host for plasmid manipulation | Novagen |
| DH5α | Cloning host for plasmid manipulation | Novagen |
| BL21 (DE3) | Host for protein expression | Novagen |
| ET12567/pUZ8002 | Non-methylating ET12567 containing non-transmissible RP4 derivative plasmid pUZ8002 | (Kieser et al., 2000) |
| Plasmids | | |
| pBluescript II (KS+) | Routine cloning and subcloning vector | Novagen |
| pSET152 | Integrative *E. coli*-*Streptomyces* shuttle vector | (Kieser et al., 2000) |
| pKCcas9dO | A CRISPR/Cas9 editing plasmid, pSG5, *tipA*-*Scocas9*, j23119, *actII-orf4* guide-RNA, homologous region flanking *actII-orf4* | (Huang et al., 2015) |
| pSET-dCas9 | pSET152 carrying the dCas9 expression cassette | (Zhao et al., 2018) |
| pET-23b (+) | Vector for His-tagged protein expression in *E. coli* | Novagen |
| pGEX-4T-1 | Vector for GST-tagged protein expression in *E. coli* | GE Healthcare |
| pKCcas9dscyA1 | Derived from pKCcas9dO, *scyA1* guide-RNA, homologous region flanking *scyA1* | This study |
| pKCcas9dscyR1 | Derived from pKCcas9dO, *scyR1* guide-RNA, homologous region flanking *scyR1* | This study |
| pKCcas9dscyR2 | Derived from pKCcas9dO, *scyR2* guide-RNA, homologous region flanking *scyR2* | This study |
| pKCcas9dscyA3 | Derived from pKCcas9dO, *scyA3* guide-RNA, homologous region flanking *scyA3* | This study |
| pKCcas9dscyR3 | Derived from pKCcas9dO, *scyR3* guide-RNA, homologous region flanking *scyR3* | This study |
| pSET-dCas9-*scyR1* | Derived from pSET-dCas9, *scyR1* guide-RNA | This study |
| pSET152-P_hrdB_scyA1 | pSET152 containing one copy of *scyA1* driven by *hrdB* promoter | This study |
| pSET152-P_hrdB_scyR1 | pSET152 containing one copy of *scyR1* driven by *hrdB* promoter | This study |
| pSET152-P_hrdB_scyR2 | pSET152 containing one copy of *scyR2* driven by *hrdB* promoter | This study |
| pSET152-P_hrdB_scyA3 | pSET152 containing one copy of *scyA3* driven by *hrdB* promoter | This study |
| pSET152-P_hrdB_scyR3 | pSET152 containing one copy of *scyR3* driven by *hrdB* promoter | This study |
| pET-23b::scyR1 | *scyR1* expression vector based on pET-23b | This study |
| pET-23b::scyR3 | *scyR3* expression vector based on pET-23b | This study |
| pGEX-4T-1::scyR2 | *scyR2* expression vector based on pGEX-4T-1 | This study |
| pSET152::P*_sbbA_gfp*::SF14*sbbR* | pSET152 derivative; containing P_sbbA_-driven *gfp* and P_SF14_-driven *sbbR* | (He et al., 2018) |
| pSET152::P_A1_*gfp* | pSET152 derivative; containing P_A1_-driven *gfp* | This study |
| pSET152::P_A1_*gfp*::SF14*scyR1* | pSET152::P_A1_*gfp* derivative; containing P_SF14_-driven *scyR1* | This study |

##

## Supplementary Table 2. Primers used in this study.

| Primers | Sequence (5’-3’)^a^ | Usage |
| --- | --- | --- |
| **For disruption, complementation and overexpression** | | |
| CRISPR-scyA1LF | **TGAAAAAGTGGCACCGAGTCGGTGCTTTTTTTGAG**GACCAAAATCGCCTGCCGC | Deletion of *scyA1* gene |
| CRISPR-scyA1LR | GCCGATCCATTCGTGGAGGG |  |
| CRISPR-scyA1RF | CACACCCAACTCCAAGCCACC |  |
| CRISPR-scyA1RR | TCCCAAGCTTGATCAAGACACGGACCCTACGC |  |
| scyA1sgRNAF | GGACTAGT*TCGCCAAAGCCCACATCACC*GTTTTAGAGCTAGAAA |  |
| scyA1sgRNAR | CTCAAAAAAAGCACCGACTCGG |  |
| VscyA1F | CCTGATCCACCGTCAACCG | Confirmation of *scyA1* deletion in ΔscyA1 |
| VscyA1R | GGACTTGCTGTCCGATCCCT |  |
| CRISPR-scyR1LF | **TGAAAAAGTGGCACCGAGTCGGTGCTTTTTTTGAG**CCAGGCGGAGTCTTCGGTAGGA | Deletion of *scyR1* gene |
| CRISPR-scyR1LR | GACCAAAATCGCCTGCCGC |  |
| CRISPR-scyR1RF | TATGAGGAGGCGGTCGGTGC |  |
| CRISPR-scyR1RR | TCCCAAGCTTCCACCAGCCAGCAGCCCATAA |  |
| scyR1sgRNAF | GGACTAGT*GGTGCTGGAGGAGGCGAAGG*GTTTTAGAGCTAGAAA |  |
| scyR1sgRNAR | CTCAAAAAAAGCACCGACTCGG |  |
| VscyR1F | CGAGGAGGCTGCCGAGGAAGAG | Confirmation of *scyR1* deletion in ΔscyR1 |
| VscyR1R | CACCAGACCACCGCACCAGACC |  |
| CRISPR-scyR2LF | **TGAAAAAGTGGCACCGAGTCGGTGCTTTTTTTGAG**GCGCAGTGTCTCCACCCAGAG | Deletion of *scyR2* gene |
| CRISPR-scyR2LR | GTAGACGGAGCCGCTGGTCC |  |
| CRISPR-scyR2RF | CGCGCCTTCTTCGGCCTGTG |  |
| CRISPR-scyR2RR | TCCCAAGCTTTACGTGGCGGCGTTCAGC |  |
| scyR2sgRNAF | GGACTAGT*CGCCGGGCACCTCGCCCGCC*GTTTTAGAGCTAGAAA |  |
| scyR2sgRNAR | CTCAAAAAAAGCACCGACTCGG |  |
| VscyR2F | CGGCGACTTCGGCAGCAACC | Confirmation of *scyR2* deletion in ΔscyR2 |
| VscyR2R | CTGGCAGTGCTCGGACATCACCG |  |
| CRISPR-scyA3LF | **TGAAAAAGTGGCACCGAGTCGGTGCTTTTTTTGAG**GGGACATCAATCCTCCGTGAGC | Deletion of *scyA3* gene |
| CRISPR-scyA3LR | CGGGTGGTAGAAGGCGTGCTCG |  |
| CRISPR-scyA3RF | ACCGAGTTCGACGAGCCCTG |  |
| CRISPR-scyA3RR | TCCCAAGCTTCGTCGTCATCGACAACTCCTCC |  |
| scyA3sgRNAF | GGACTAGT*CGCCCCGGGCATGCTCCTGC*GTTTTAGAGCTAGAAA |  |
| scyA3sgRNAR | CTCAAAAAAAGCACCGACTCGG |  |
| VscyA3F | TCGTCCAGCGAGCCGACCA | Confirmation of *scyA3* deletion in ΔscyA3 |
| VscyA3R | GCACAGCCGTCCTCCCCTCA |  |
| CRISPR-scyR3LF | **TGAAAAAGTGGCACCGAGTCGGTGCTTTTTTTGAG**TTCGGCTTCGCTCACAGGC | Deletion of *scyR3* gene |
| CRISPR-scyR3LR | CGATTAGCTCCTCCAAAGTAGAACG |  |
| CRISPR-scyR3RF | AACCGGCGCCTCAGGGCC |  |
| CRISPR-scyR3RR | TCCCAAGCTTGCGTAACCGGCGTTGTTGAAGG |  |
| scyR3sgRNAF | GGACTAGT*CTTCGATTCCAAGGAGGACC*GTTTTAGAGCTAGAAA |  |
| scyR3sgRNAR | CTCAAAAAAAGCACCGACTCGG |  |
| VscyR3F | GCCTCGCCCTGTTCATCTCC | Confirmation of *scyR3* deletion in ΔscyR3 |
| VscyR3R | CAGCACCTGCTCCATCATCTCC |  |
| CRISPRi-scyR1sgRNAF | TTGGACTAGT*GCGTACGCGTCGCACGTTCC*GTTTTAGAGCTAGAAATA | Repression of *scyR1* gene |
| CRISPRi-scyR1sgRNAR | TAGAATTCGGGTGTACATCCAGTAATG |  |
| hrdB-pF | AATTTCTAGACCGCCTTCCGCCGGAACG | *hrdB* promoter |
| hrdB-pR | GAACAACCTCTCGGAACGTTG |  |
| OEscyA1F | **CCACAACGGTTTCCCTCTAG**GAATTCATGACCCTGCTGACGCTCCACCA | Overexpression of *scyA1* in NMWT1; complementation of *scyA1* in ΔscyA1 |
| OEscyA1R | **TGGTGGTGGTGGTGCTCGAG**GAATTCTTATGGGGTGTGGGTTGTGGGGTG |  |
| OEscyR1F | **CCACAACGGTTTCCCTCTAG**GAATTCGTGGCGCGCCAGGAACGTG | Overexpression of *scyR1* in NMWT1; overexpression of *scyR1* in ΔscyA1 |
| OEscyR1R | **TGGTGGTGGTGGTGCTCGAG**GAATTCTCACCCGGTCACCGCGGGA |  |
| OEscyR2F | **CCACAACGGTTTCCCTCTAG**GAATTCGTGGACAGCACGGTGCAGGAACG | Overexpression of *scyR2* in NMWT1 |
| OEscyR2R | **TGGTGGTGGTGGTGCTCGAG**GAATTCTCAGGCGGTGGCTCCGGAGG |  |
| OEscyA3F | **CCACAACGGTTTCCCTCTAG**GAATTCATGAGTGCAGTGTTCCAGCAGGGAT | Overexpression of *scyA3* in NMWT1 |
| OEscyA3R | **TGGTGGTGGTGGTGCTCGAG**GAATTCTCACAGGCCCGGCGCCGAC |  |
| OEscyR3F | **CCACAACGGTTTCCCTCTAG**GAATTCATGGCGGGCGGCGTGAAG | Overexpression of *scyR3* in NMWT1 |
| OEscyR3R | **TGGTGGTGGTGGTGCTCGAG**GAATTCTCAGTCCAGCGGGGTCAGCG |  |
| **For protein expression** | | |
| PscyR1F | GGAATTCCATATGGCGCGCCAGGAACGTGCG | Overexpression of His_6_-tagged ScyR1 in *E.coli* |
| PscyR1R | CCGCTCGAGCCCGGTCACCGCGGGAGCCAG |  |
| PscyR3F | GGAATTCCATATGGCGGGCGGCGTGAAGCA | Overexpression of His_6_-tagged ScyR3 in *E.coli* |
| PscyR3R | CCGCTCGAGGTCCAGCGGGGTCAGCGCGC |  |
| PscyR2F | CGGAATTCGTGGACAGCACGGTGCAGGA | Overexpression of GST-tagged ScyR2 in *E.coli* |
| PscyR2R | CCGCTCGAGGGCGGTGGCTCCGGAGGCAA |  |
| **For EMSAs** | | |
| PhrdBF | AATTTCTAGACCGCCTTCCGCCGGAACG | Probe P_hrdB_ |
| PhrdBR | GAACAACCTCTCGGAACGTTG |  |
| PscyA1F | CACCTGCTCAGCCAGCACACTCT | Probe P_A1_ |
| PscyA1R | GTGGAGGTGCCGGTATGCGG |  |
| PnemA1-1F | TTTCCTGCGTTCCGTAGTGC | Probe P_nemA1-1_ |
| PnemA1-1R | CGACGGACCAGTCGAGGTAC |  |
| PnemA4F | CCAGTTCGGCGGTCGCCTCC | Probe P_nemA4_ |
| PnemA4R | CGACCAGCTCCCACAGGTCC |  |
| PnemCF | GCGGTGGGCGAGAAGGAGCT | Probe P_nemC_ |
| PnemCR | GCACCTGCGGTAGAGCCACA |  |
| PnemRF | GATGCCGCCGCGTGCTTGAG | Probe P_nemR_ |
| PnemRR | CGGTCCTGTCGTCCGGGGTA |  |
| **For qRT-PCR** | | |
| q-16sF | CCTTACCAAGGCTTGACATACA | *16s* ORF |
| q-16sR | CGGGACTTAACCCAACATCTC |  |
| q-scyA1F | CAGCATCCACTACAACCTCTTC | *scyA1* ORF |
| q-scyA1R | GGAAGGTGCCGGTGTTT |  |
| q-scyR1F | TGCTGTCGAACATCATGACC | *scyR1* ORF |
| q-scyR1R | CCGGAGCGAAATCCATCTG |  |
| q-scyR2F | AACCCGCAGGTCTCTTCT | *scyR2* ORF |
| q-scyR2R | TGCTGGCGTAGTGGAAGTA |  |
| q-scyA3F | CCGCCGAACTCGAACTG | *scyA3* ORF |
| q-scyA3R | GTCCGTTGAGCGTGATGT |  |
| q-scyR3F | TCTTCGACGAGTTCGGGTA | *scyR3* ORF |
| q-scyR3R | CCTTGGAATCGAAGTGGAAGT |  |
| q-nemRF | TCGAAGAACTCGTCGAAAGC | *nemR* ORF |
| q-nemRR | AGGACGGCCTGGTAGAA |  |
| q-nemA1-2F | CTTCTTCGGGATCTCCCCGC | *nemA1-2* ORF |
| q-nemA1-2R | GTACCGAGGACGGGTCGATG |  |
| q-Tu94_00870F | GGTCGCCTTCATGTTCACCG | *Tu94_00870* ORF |
| q-Tu94_00870R | TCGACGACCTGCCTGAACAA |  |
| q-Tu94_02330F | TCTGCCACGACCTCGATCTG | *Tu94_02330* ORF |
| q-Tu94_02330R | GAGTTCGACCCCATCAGCCC |  |
| q-Tu94_02965F | CCCCTGTTCAGCTCGGTGAT | *Tu94_02965* ORF |
| q-Tu94_02965R | AGACCGTCAGCGGGTAGTTG |  |
| q-Tu94_04235F | CCCCGTTCGTCGTCCTCTG | *Tu94_04235* ORF |
| q-Tu94_04235R | CTGTTCGGACTCGGGCAGAT |  |
| q-Tu94_04905F | ACATCGGCTACCTGTGCTGT | *Tu94_04905* ORF |
| q-Tu94_04905R | TGACGGGGATGCCGAGTTC |  |
| q-Tu94_11785F | TACCCCACCCCGCTGGA | *Tu94_11785* ORF |
| q-Tu94_11785R | TGTACACCATGGCGGTCTCC |  |
| q-Tu94_21765F | CGGAAACGGTGCGGAAACAT | *Tu94_21765* ORF |
| q-Tu94_21765R | TTGGGCAGTGAGCAGAGGTC |  |
| q-Tu94_22200F | TCGAACCACCCGACATCGAC | *Tu94_22200* ORF |
| q-Tu94_22200R | CCGGAAGCCCATCGTGTTGA |  |
| q-Tu94_22430F | CACCTTCGACCTGGTGGTCA | *Tu94_22430* ORF |
| q-Tu94_22430R | ACTGCCGTACGAAGGTGTCG |  |
| q-Tu94_23005F | GTTCGGGGTCATGGTCGAGT | *Tu94_23005* ORF |
| q-Tu94_23005R | TACAGCGTGAGGTCGAACGG |  |
| q-Tu94_27405F | AGACCAAACGGCTCACCGAC | *Tu94_27405* ORF |
| q-Tu94_27405R | GTGTCATGCCCTCGCTGGT |  |
| q-Tu94_29090F | GGGACGAGCCCTACTTCACC | *Tu94_29090* ORF |
| q-Tu94_29090R | GGGCTCTCCGTTCACGTACC |  |
| q-Tu94_29680F | CATACACGCCACGAACGAGC | *Tu94_29680* ORF |
| q-Tu94_29680R | AGGACACCGTTTCCACCGTT |  |
| q-Tu94_30615F | TGAAGGCGTACACCGGACTG | *Tu94_30615* ORF |
| q-Tu94_30615R | TTGGTGGTCTCGATGCCGTC |  |
| q-Tu94_31495F | CATCAACGCCCTGAACACCG | *Tu94_31495* ORF |
| q-Tu94_31495R | TTCAGGCCCGTGACACCC |  |
| q-Tu94_31830F | CGAGTTCTCCCACCGCGAA | *Tu94_31830* ORF |
| q-Tu94_31830R | GGATGGAGATCTGGCCGGTG |  |
| q-Tu94_32305F | ATGATGGCGCATCTCACCGA | *Tu94_32305* ORF |
| q-Tu94_32305R | GTCGAAGAGGGCCAAACCCT |  |
| **For GFP reporter system** | | |
| pscyA1GFPF | **GAATTCGATATCGCGCGCGGCCGCG**CAAAACCACCTCCGCAACCCC | *scyA1* promoter |
| pscyA1GFPR | **TGAACAGCTCTTCGCCTTTACGCAT**GGGGCCTCTTCCGCTGGGTC |  |
| GFPF | ATGCGTAAAGGCGAAGAGCTGTT | Green fluorescence gene (*gfp*) |
| GFPR | **GCCAAGCTTGGGCTGCAGGTCGACT**TCATTTGTACAGTTCATCCATACCATGC |  |
| ScyR1GFPF | GTGGCGCGCCAGGAACGTG | *scyR1* |
| ScyR1GFPR | **GATCCCCGGGGACCTGCAGGTCGACTCTAG**TCACCCGGTCACCGCGGG |  |
| pSF14F | **ACTCACCGCGACGTATCGGGCCCTGGCCAG**GCCTTGACCTTGATGAGGCG | SF14 promoter |
| pSF14R | **CGTACGCGTCGCACGTTCCTGGCGCGCCAC**CTAATCGAGTATTGATTGTAGCTCACG |  |

^a^ The introduced restriction sites are underlined. The overlapped sequences used for overlap PCR or one step cloning are in bold. The guide sequence of sgRNA used in gene deletion construction is shown in italic.

## Supplementary Table 3. BlastP analysis of ScyR1, ScyR2, ScyR3 and their homologs. Percentage coverage/identify values of the three γ-butyrolactone (GBL) receptor homologs in NMWT1 to those of *S. griseus*, *S. coelicolor*, *S. venezuelae*, *S. avermitilis* and *S. fradiae*, are given in the table.

| Protein | Coverage/identity * (%) to genes of | | | | |
| --- | --- | --- | --- | --- | --- |
| *S. cyaneogriseus* ssp*. noncyanogenus* NMWT1 | *S.*  *griseus* (Khokhlov et al., 1967) | *S. coelicolor* (Takano et al., 2001) | *S. venezuelae* (Zou et al., 2014) | *S. avermitilis* (Kitani et al., 2011) | *S.*  *fradiae* (Bignell et al., 2007) |
|  | ArpA | ScbR | JadR3 | AvaR1 | TylP |
| ScyR1  (TU94_00975) | 90/37 | 91/45 | 88/46 | 92/70 | 96/60 |
| ScyR2  (TU94_03165) | 85/31 | 85/30 | 85/33 | 85/31 | 81/33 |
| ScyR3  (TU94_11460) | 91/43 | 94/43 | 95/39 | 97/41 | 97/45 |

## Supplementary Table 4. BlastP analysis of ScyA1, TU94_00970, ScyA3 and their homologs. Percentage coverage/identify values of ScyA1, TU94_00970, ScyA3 to those of *S*. *avermitilis*, *S*. *fradiae* and *S*. *aibus* J1074 are given in parentheses.

| Protein | Coverage/identity * (%) to genes of | | | Putative function |
| --- | --- | --- | --- | --- |
| *S. cyaneogriseus* ssp. *noncyanogenus* NMWT1 | *S. avermitilis* (Kitani et al., 2011) | *S. fradiae*  (Bignell et al., 2007) | *S. albus* J1074 (Ahmed et al., 2017) |  |
| ScyA1  (TU94_00985) | Aco  (64/63) | Orf18*  (86/51) | XNR_2339  (85/47) | acyl-CoA oxidase |
| TU94_00970 | Cyp17  (98/70) | Orf16*  (100/66) | XNR_2340  (94/30) | cytochrome P450 |
| ScyA3  (TU94_11455) | SAV_2269  (85/32) | D3X13_12515  (71/33) | - | gamma-butyrolactone biosynthesis protein |

- not found**.**

## Supplementary Table 5. 17 secondary metabolite biosynthetic core genes and the corresponding associated biosynthetic gene cluster in *S. cyaneogriseu*s ssp. *noncyanogenus* NMWT1.

| # | Biosynthetic core gene | Putative function | Type | Most similar known cluster | Similarity (%) |
| --- | --- | --- | --- | --- | --- |
| 01 | *Tu94_00870* | [type I polyketide synthase](https://blast.ncbi.nlm.nih.gov/Blast.cgi#alnHdr_WP_078969450) | T1PKS-NRPS | Merochlorin biosynthetic gene cluster | 19 |
| 02 | *Tu94_02330* | [type II polyketide synthase](https://blast.ncbi.nlm.nih.gov/Blast.cgi#alnHdr_WP_078969450) | T2PKS | Spore pigment biosynthetic gene cluster | 83 |
| 03 | *Tu94_02965* | [non-ribosomal peptide synthetase](https://blast.ncbi.nlm.nih.gov/Blast.cgi#alnHdr_WP_044378968) | T1PKS-NRPS | Tautomycin biosynthetic gene cluster | 27 |
| 04 | *Tu94_04235* | [non-ribosomal peptide synthetase](https://blast.ncbi.nlm.nih.gov/Blast.cgi#alnHdr_WP_078969061) | NRPS | Azicemicin biosynthetic gene cluster | 8 |
| 05 | *Tu94_04905* | [type III polyketide synthase](https://blast.ncbi.nlm.nih.gov/Blast.cgi#alnHdr_WP_078969450) | T3PKS | Pheganomycin biosynthetic gene cluster | 28 |
| 06 | *Tu94_11785* | [tyrosinase](https://blast.ncbi.nlm.nih.gov/Blast.cgi#alnHdr_WP_053662318) | Melanin | Melanin biosynthetic gene cluster | 60 |
| 07 | *Tu94_21765* | [terpene synthase](https://blast.ncbi.nlm.nih.gov/Blast.cgi#alnHdr_BAP82219) | Terpene | Albaflavenone biosynthetic gene cluster | 100 |
| 08 | *Tu94_22200* | [type III polyketide synthase](https://blast.ncbi.nlm.nih.gov/Blast.cgi#alnHdr_WP_078969450) | T3PKS | Naringenin biosynthetic gene cluster | 100 |
| 09 | *Tu94_22430* | [LuxR family transcriptional regulator](https://blast.ncbi.nlm.nih.gov/Blast.cgi#alnHdr_WP_078969287) | T1PKS | Oligomycin biosynthetic gene cluster | 88 |
| 10 | *Tu94_23005* | non-ribosomal peptide synthetase | T1PKS-NRPS | BD-12 biosynthetic gene cluster | 17 |
| 11 | *Tu94_27405* | [terpene synthase family protein](https://blast.ncbi.nlm.nih.gov/Blast.cgi#alnHdr_WP_044385555) | Terpene | Pentalenolactone biosynthetic gene cluster | 58 |
| 12 | *Tu94_29090* | [squalene--hopene cyclase](https://blast.ncbi.nlm.nih.gov/Blast.cgi#alnHdr_WP_044386119) | Terpene | Hopene biosynthetic gene cluster | 84 |
| 13 | *Tu94_29680* | transAT- polyketide synthase | T1PKS-NRPS | Reveromycin biosynthetic gene cluster | 9 |
| 14 | *Tu94_30615* | [non-ribosomal peptide synthetase](https://blast.ncbi.nlm.nih.gov/Blast.cgi#alnHdr_WP_078969417) | NRPS | Taromycin biosynthetic gene cluster | 26 |
| 15 | *Tu94_31495* | type A2 lantipeptide | Bacteriocin | Informatipeptin biosynthetic gene cluster | 42 |
| 16 | *Tu94_31830* | [type II polyketide synthase](https://blast.ncbi.nlm.nih.gov/Blast.cgi#alnHdr_WP_078969450) | T2PKS | Enterocin biosynthetic gene cluster | 85 |
| 17 | *Tu94_32305* | [type I polyketide synthase](https://blast.ncbi.nlm.nih.gov/Blast.cgi#alnHdr_WP_078969450) | T3PKS-NRPS | Pheganomycin biosynthetic gene cluster | 42 |

# Supplementary References

Ahmed, Y., Rebets, Y., Tokovenko, B., Brotz, E., and Luzhetskyy, A. (2017). Identification of butenolide regulatory system controlling secondary metabolism in *Streptomyces albus* J1074. *Sci. Rep.* 7, 9784. doi: 10.1038/s41598-017-10316-y

Bignell, D. R., Bate, N., and Cundliffe, E. (2007). Regulation of tylosin production: role of a TylP-interactive ligand. *Mol. Microbiol.* 63, 838-847. doi: 10.1111/j.1365-2958.2006.05541.x

He, H., Ye, L., Li, C., Wang, H., Guo, X., Wang, X. et al. (2018). SbbR/SbbA, an important ArpA/AfsA-like system, regulates milbemycin production in *Streptomyces bingchenggensis*. *Front. Microbiol.* 9, 1064. doi: 10.3389/fmicb.2018.01064

Huang, H., Zheng, G., Jiang, W., Hu, H., and Lu, Y. (2015). One-step high-efficiency CRISPR/Cas9-mediated genome editing in *Streptomyces*. *Acta. Biochim. Biophys. Sin. (Shanghai).* 47, 231-243. doi: 10.1093/abbs/gmv007

Khokhlov, A. S., Tovarova, I. I., Borisova, L. N., Pliner, S. A., and Rapoport, I. A. (1967). The A-factor, responsible for streptomycin biosynthesis by mutant strains of *Actinomyces streptomycini*. *Dokl. Akad. Nauk SSSR* 177, 232-235.

Kieser, T., Bibb, M. J., Buttner, M. J., Chater, K. F., Hopwood, D. A., Charter, K. et al. (2000). Practical *Streptomyces* Genetics. Norwich: The John Innes Foundation.

Kitani, S., Miyamoto, K. T., Takamatsu, S., Herawati, E., Iguchi, H., Nishitomi, K. et al. (2011). Avenolide, a *Streptomyces* hormone controlling antibiotic production in *Streptomyces avermitilis*. *Proc. Natl. Acad. Sci. U S A* 108, 16410-16415. doi: 10.1073/pnas.1113908108

Takano, E., Chakraburtty, R., Nihira, T., Yamada, Y., and Bibb, M. J. (2001). A complex role for the gamma-butyrolactone SCB1 in regulating antibiotic production in *Streptomyces coelicolor* A3(2). *Mol. Microbiol.* 41, 1015-1028. doi: 10.1046/j.1365-2958.2001.02562.x

Zhao, Y., Li, L., Zheng, G., Jiang, W., Deng, Z., Wang, Z. et al. (2018). CRISPR/dCas9-mediated multiplex gene repression in *Streptomyces*. *Biotechnol. J.* 13, e1800121. doi: 10.1002/biot.201800121

Zou, Z., Du, D., Zhang, Y., Zhang, J., Niu, G., and Tan, H. (2014). A gamma-butyrolactone-sensing activator/repressor, JadR3, controls a regulatory mini-network for jadomycin biosynthesis. *Mol. Microbiol.* 94, 490-505. doi: 10.1111/mmi.12752
